# Different internal fixation methods for Hoffa-like fractures of the tibial plateau: a finite element analysis

**DOI:** 10.3389/fmed.2023.1172377

**Published:** 2023-07-03

**Authors:** Hang Xue, Junrong Deng, Zhenhe Zhang, Samuel Knoedler, Adriana C. Panayi, Leonard Knoedler, Bobin Mi, Mengfei Liu, Guandong Dai, Guohui Liu

**Affiliations:** ^1^Department of Orthopedics, Jingshan Union Hospital, Union Hospital, Huazhong University of Science and Technology, Wuhan, China; ^2^Division of Plastic Surgery, Department of Surgery, Brigham and Women's Hospital, Harvard Medical School, Boston, MA, United States; ^3^Department of Plastic, Hand and Reconstructive Surgery, University Hospital Regensburg, Regensburg, Germany; ^4^Pingshan District People’s Hospital of Shenzhen, Pingshan General Hospital of Southern Medical University, Shenzhen, China

**Keywords:** finite element analysis, tibial plateau fracture, Hoffa fracture, posteromedial split, internal fixation

## Abstract

Due to the low incidence of posteromedial tibial plateau fractures and limited clinical data available, the optimal treatment for this type of fracture remains to be established. This type of fracture, also known as Hoffa-like fracture of the tibial plateau, shares a similar mechanism of injury with the Hoffa fracture of the femoral condyle. In the field of orthopedics, finite element analysis is considered a valuable method to guide clinical decision-making. In this study, four methods used for internal fixation of Hoffa-like fractures of the tibial plateau were compared using computer simulation and applying a finite element method (FEM). The methods compared were lateral L-plate fixation alone (Model A); lateral L-plate combined with posterior anti-slip plate (reconstruction plate/T-plate) fixation (Model B); lateral L-plate combined with posterior hollow nail fixation of the fracture block (Model C); and lateral L-plate combined with anterior hollow nail fixation of the fracture (Model D). The maximum displacement of the model and the maximum stress of the internal fixation material were analyzed by applying an axial load of 2,500 N. The results showed that, in the normal bone model, the maximum displacement of the fracture in Model A was 0.60032 mm, with improved stability through the addition of posterior lateral plate fixation in Model B and reduction of the displacement to 0.38882 mm. The maximum displacement in Model C and Model D was comparable, amounting to 0.42345 mm and 0.42273 mm, respectively. Maximum stress was 1235.6 MPa for Model A, 84.724 MPa for Model B, 99.805 MPa for Model C, and 103.19 MPa for Model D. In the internal fixation analysis of the osteoporotic fracture model, we observed patterns similar to the results of the normal bone model. The results indicated that Model B yielded the overall best results in the treatment of Hoffa-like fractures of the tibial plateau. The orthopedic surgeon may wish to implement these insights into the perioperative algorithm, thereby refining and optimizing clinical patient care. In addition, our findings pave the way for future research efforts.

## 1. Introduction

The most common forms of intra-articular fractures of the knee are distal femoral and tibial plateau fractures, both of which account for more than 80% of all cases (1). The Hoffa fracture, a type of coronal fracture of the femoral condyle, is a rare clinical fracture, representing only 0.1% of all fracture types. This type of fracture was first described by Hoffa in 1888 and, thus, named Hoffa fracture (2). It is widely accepted among orthopedic surgeons that Hoffa fractures of the femoral condyles occur by a mechanism primarily due to the result of axial force. Based on the concept of action and reaction forces, there is a mounting body of evidence indicating that the same mechanism of injury is associated with Hoffa-like fractures of the tibial plateau (3, 4). In fact, this type of fracture was commonly referred to as a coronal split fracture of the tibial plateau in previous case reports and was subsequently linked to the Hoffa fracture of the femoral condyle (4–6). Zhu et al. successfully simulated this fracture type in biomechanical experiments and re-named it Hoffa-like fracture of the tibial plateau due to the same injury mechanism as the Hoffa fracture of the femoral condyle (1). Due to its rare incidence, the Hoffa-like fracture of the tibial plateau is not classified in either the Schatzker or AO/OTA classification systems (3, 4). Chang et al. documented a case series of posterior coronal fractures of the tibial plateau with a collapsed fracture, possibly due to different age groups or varying mechanisms of injury (3).

A growing number of orthopedic surgeons have acknowledged the importance of considering posterior medial fracture fixation in tibial plateau fractures: while Luo et al. reported a method of columnar staging (i.e., the three-columnar staging of the tibial plateau based on the determination of fracture classification by CT scan), Barei et al. noted that posterior medial fractures were present in approximately one-third of cases with bicondylar fractures of the tibial plateau (7, 8). Generally, an anterolateral approach is recommended for patients with bicondylar fractures of the tibial plateau. In this context, it is worth mentioning that this surgical approach can also be applied in patients with posterior lateral column fractures (9, 10). Fractures of the coronal surface of the tibial plateau are difficult to detect on plain radiographs and, are therefore easily missed (11). Standard CT or 3D CT reconstruction can provide a definitive diagnosis of such fractures and reduce the rate of missed diagnoses. Accordingly, in cases of high-energy impact or blunt trauma, an oblique radiograph or CT scan of the knee should be promptly acquired.

Hoffa-like fractures of the tibial plateau are most frequently caused by vertical shear forces exerted from the femoral condyle on the posterior aspect of the tibial plateau when the knee is in a flexed position at the time of injury. Owing to the inherent specificity and general scarcity of these fractures, the optimal treatment modality is still controversial. Therefore, to be able to explain the biomechanical principles underlying repositioned internal fixation of Hoffa-like fractures of the tibial plateau, we developed a 3D model. This 3D simulation of the bone-endophyte structure into finite elements was based on a simplified, yet representative, model of the fracture, as well as the plates and screws required for internal fixation. In order to predict surgical outcomes, we then applied the simulated physiological loads (12–14). Using computer model simulations, finite element method (FEM) was applied to compare four different fixation methods for Hoffa-like fractures of the tibial plateau and to calculate the displacement and stress per each mechanical model.

Thus, this study aims to compare the effect of different internal fixation methods on Hoffa-like fractures of the tibial plateau. These insights may help both surgeons and patients to refine surgical decision-making.

## 2. Materials and method

### 2.1. Clinical data

This study was approved by the Ethics Committee of Union Hospital, Tongji Medical College, Huazhong University of Science and Technology. A 31-year-old male patient, 180 cm tall and weighing 80 kg, signed the informed consent form and underwent a CT scan of the left lower extremity. The Hoffa-like fracture of the tibial plateau is subject to axial forces transmitted from the femoral condyle to the posterior tibial plateau, resulting in shear-splitting fractures—which may be associated with collapse fractures in patients with osteoporosis. Therefore, the fracture line in this study was determined in the range of the posterior column which included posteromedial split and posterolateral collapse fractures.

### 2.2. Experimental model

**Figure 1:**
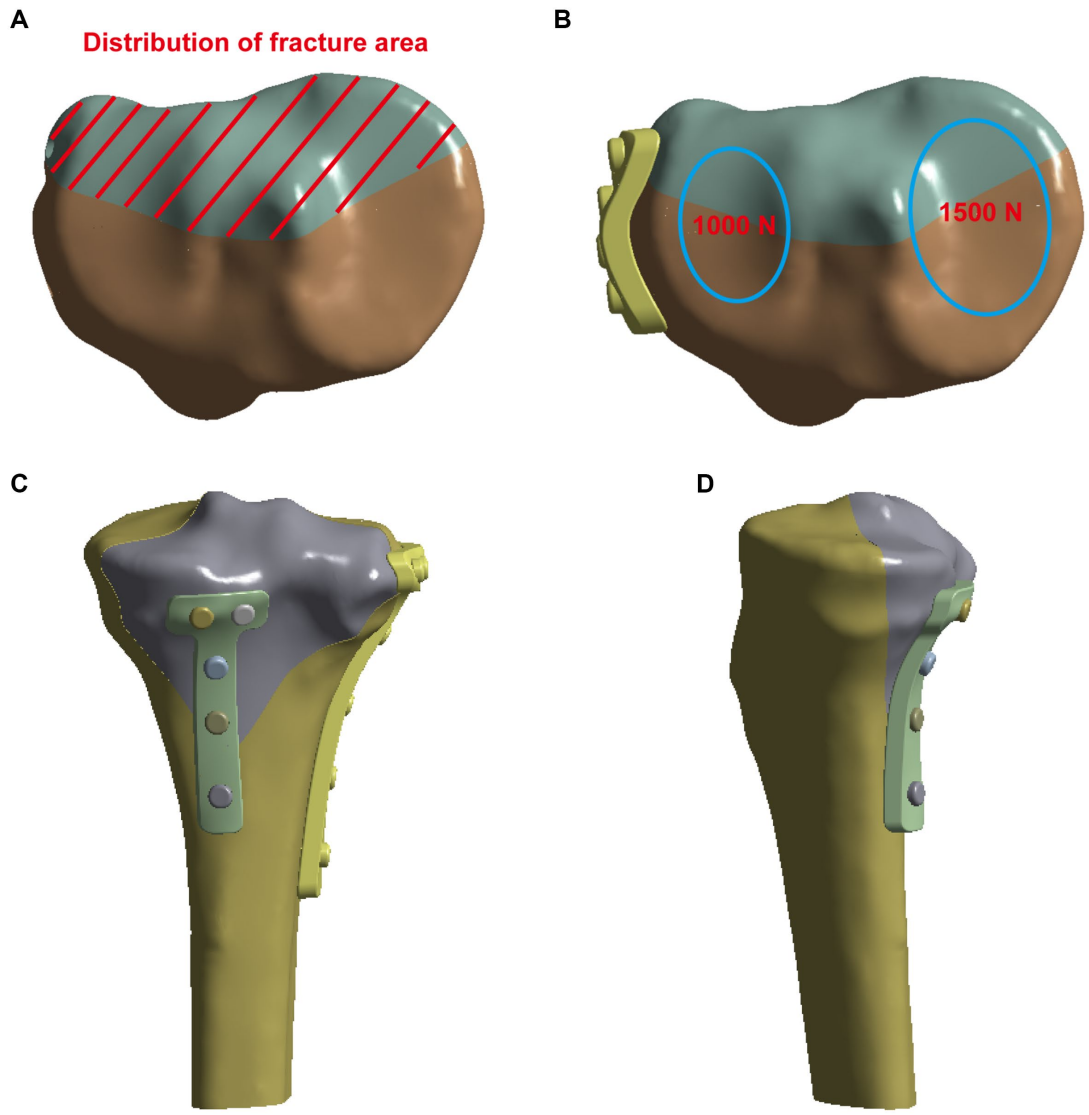
Creation of the fracture model **(A)** and the vertical force established to simulate the physiological load on the articular surface and boundary conditions **(B)**. **(C,D)** The pictures of internal fixation of fractures at different positions.

CT images of the tibia and fibula were acquired from the patient. All image data of coronal, sagittal, and axial positions were obtained. Images that met the modeling requirements were extensively scanned, with all image files being saved in the Digital Imaging and Communications in Medicine (DICOM) format. The three-dimensional (3D) model of the tibia was reconstructed by importing the CT data into Mimics 20.0 (Materialise Company, Leuven, Belgium). The reconstructed 3D model was then imported into Geomagic Studio 2015 (Raindrop Company, Marble Hill, United States) for reverse processing and create the fracture model (Figure 1).

**Figure 2:**
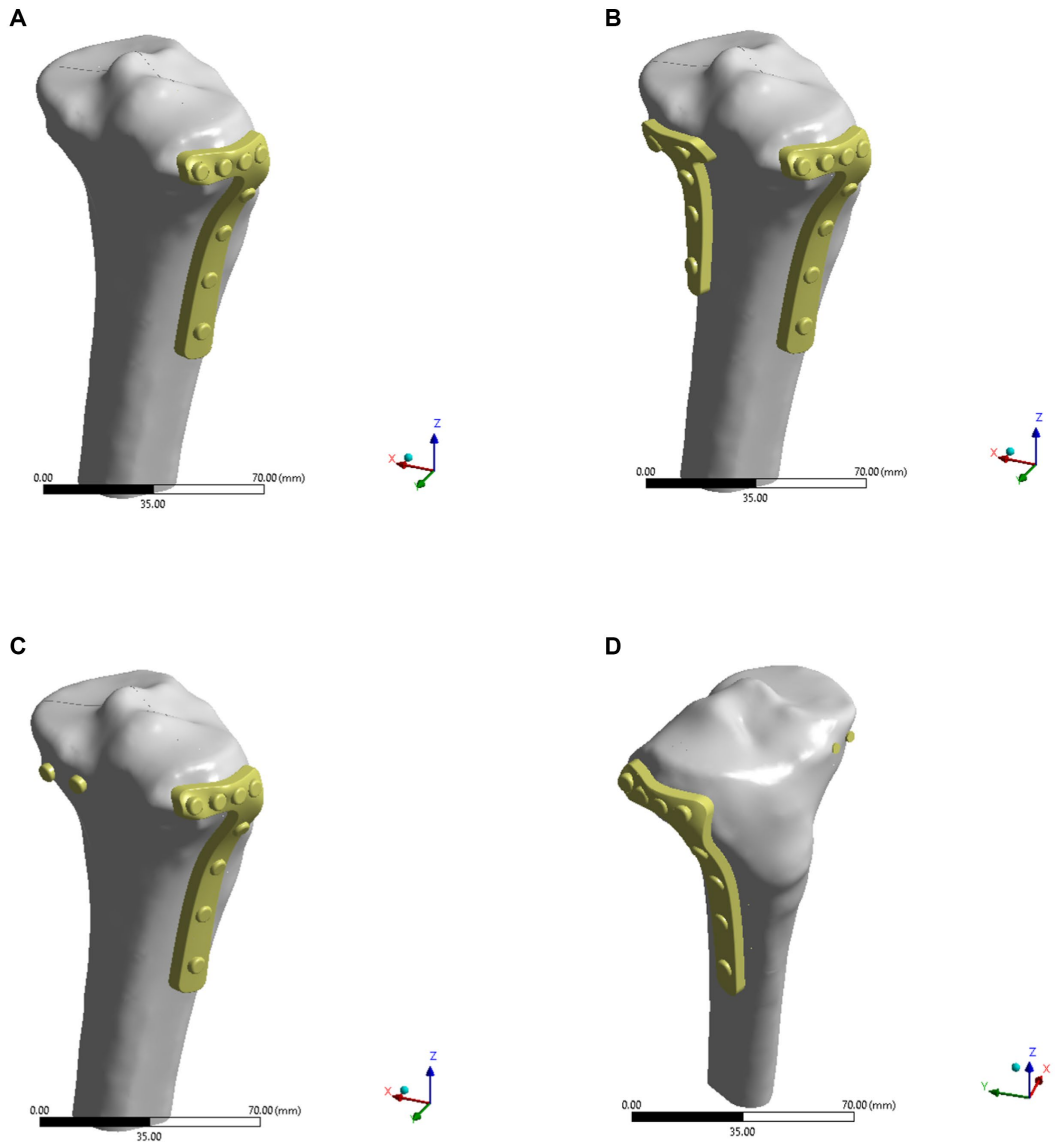
Four models of internal fixations for Hoffa-like fracture of the tibial plateau. **(A)** The lateral L-plate fixation alone in the model A. **(B)** The lateral L-plate combined with posterior anti-slip plate fixation in the model B. **(C)** The L-plate combined with posterior hollow nail fixation of the fracture block in the model C. **(D)** The L-plate combined with anterior hollow nail fixation of the fracture in the model D.

In this study, SpaceClaim software was used to prepare the analysis model. The fixation methods (and internal fixation parameters) were evaluated in four groups with different surgical schemes (Figure 2): Model A was fixation using only an L-shaped plate on the lateral tibial plateau with four nails below the knee joint and four nails along the tibial axis. Model B was fixed with an L-shaped plate and a posterior plate for tibial plateau fracture. In Model C, in addition to the L-plate fixation, two more cannulated screws were used to fix the fracture block from behind. In Model D, a lateral plate and two cannulated screws from the anterior part of the fracture block were fixed.

### 2.3. Material properties

**Figure 3:**
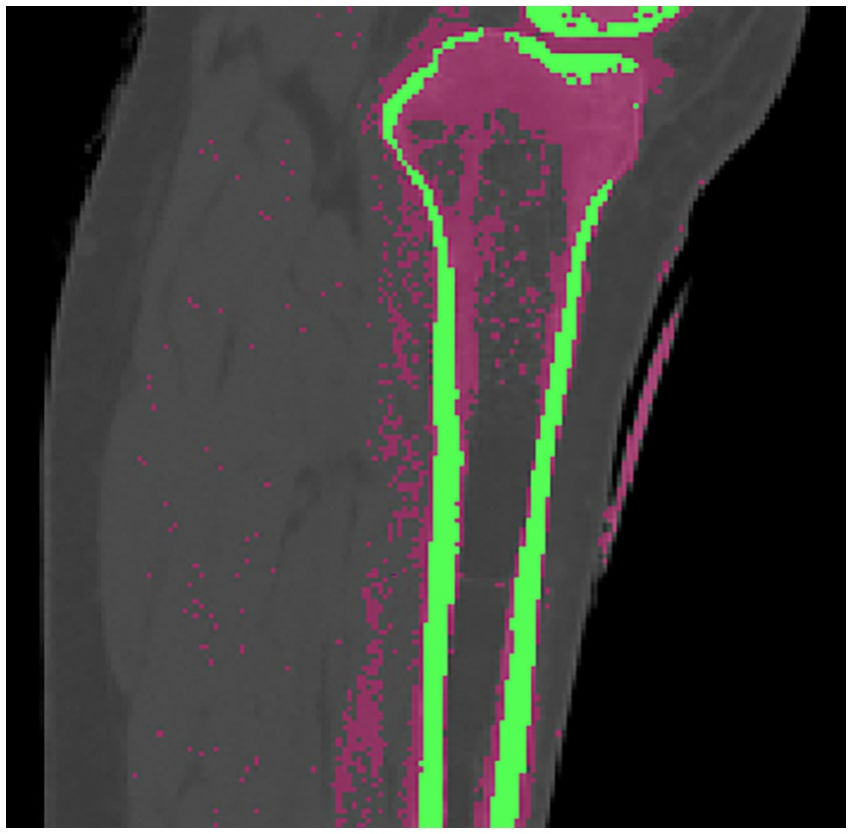
Cortical and cancellous bone were distinguished by different threshold values from the CT images, and the green part was cortical bone and the pink part was cancellous bone.

In this study, ANSYS 2021 R1 (ANSYS Company, PA, United States) was used to analyze the stresses as well as the displacements of the four fixation models in both normal bone and osteoporotic models. Four types of models were imported into ANSYS workbench and the corresponding material properties were assigned to the tibia and the internal fixation, respectively (Table 1) (14–16). In the normal bone model, Young’s modulus of cortical bone was 17 GPa and Young’s modulus of cancellous bone was 0.43 GPa. In the osteoporosis model, Young’s modulus of cortical bone and cancellous bone were 8.5 GPa and 0.215 GPa, respectively. Cortical and cancellous bone were distinguished on the basis of different threshold values from the CT images. As shown in Figure 3, cortical bone was shown in green and cancellous bone was shown in rose pink. Poisson’s ratio was 0.3 in both normal and osteoporotic bone models. After establishing contact with the model, the model was meshed. The elements global size of cortical bone and cancellous bone was set to 2 mm, plate to 1 mm, and screw to 0.5 mm, with the number of elements being 477,445 and the number of nodes 704,737. The mesh quality was then evaluated to meet the requirements of computational accuracy and convergence analysis.

**Table 1:** Material properties.

Materials	Young’s modulus (MPa)	Poisson’s ration
Cortical bone	17,000	0.3
Cancellous bone	430	0.3
Osteoporotic cortical bone	8,500	0.3
Osteoporotic cancellous bone	215	0.3
Titanium alloy plate and screw	110,000	0.3

### 2.4. Loading and boundary conditions

The bottom of the tibia was fixed, and then forces of 1,500 N and 1,000 N were exerted on the posterior side of the tibial plateau, aiming to simulate the force on the unilateral knee joint of an adult. The displacement and stress with the four fixation models using normal bone or osteoporotic bone were investigated. For plates or screws, we assumed that the contact between implant and bone is well-established and that there is a firm connection. To simulate proper contact between the plate and the screw, the interaction between the plate and the screw on the contact surface was defined as common nodes. It should be noted that FEM cannot accurately simulate the effect of reduction and internal fixation after fracture, as it does not take into account the interaction with muscles, ligaments, nerves, and vessels in the human body.

## 3. Results

**Figure 4:**
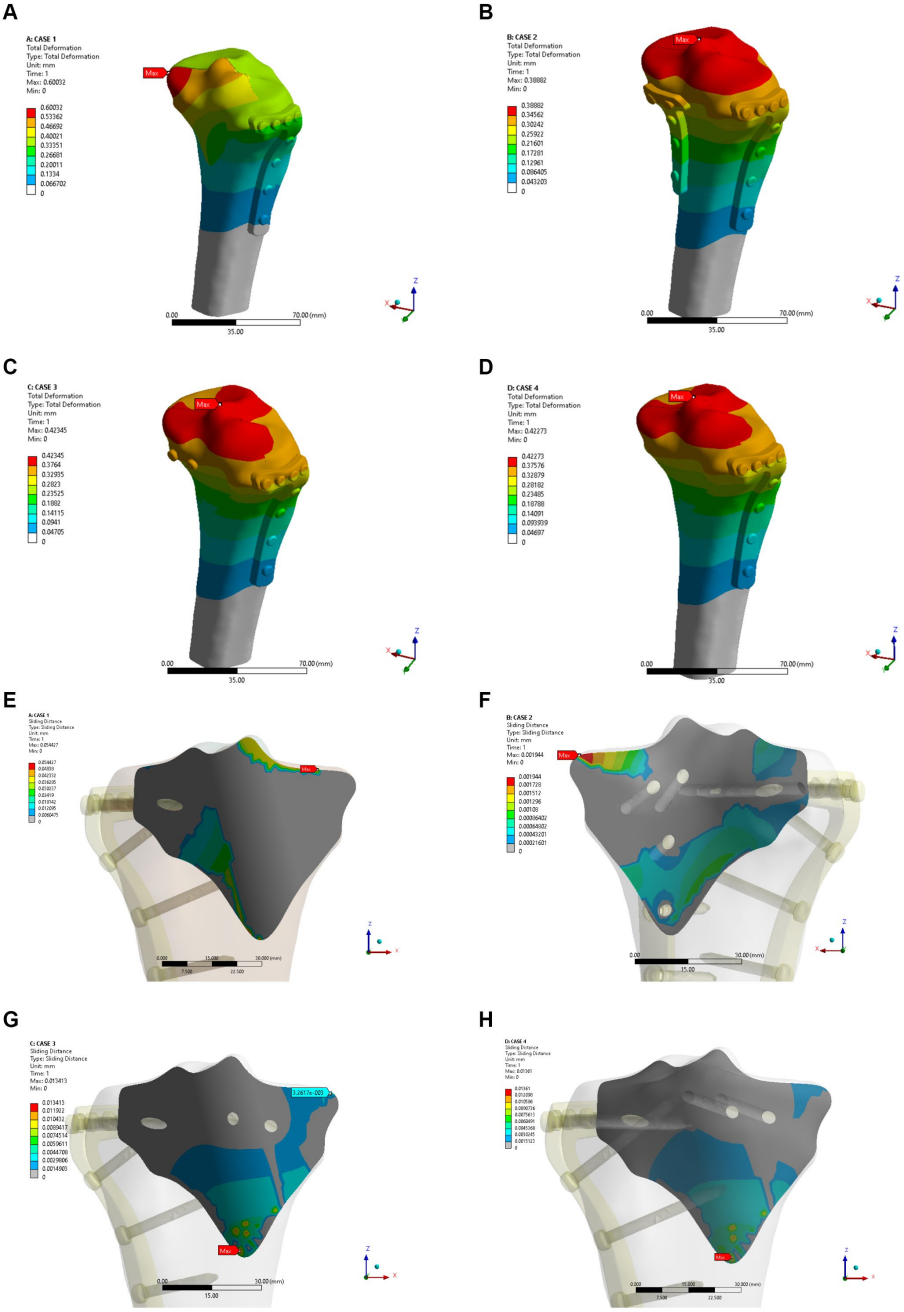
Maximum displacement **(A–D)** and interface displacement **(E–H)** in the four internal fixation models of the normal bone group.

**Figure 5:**
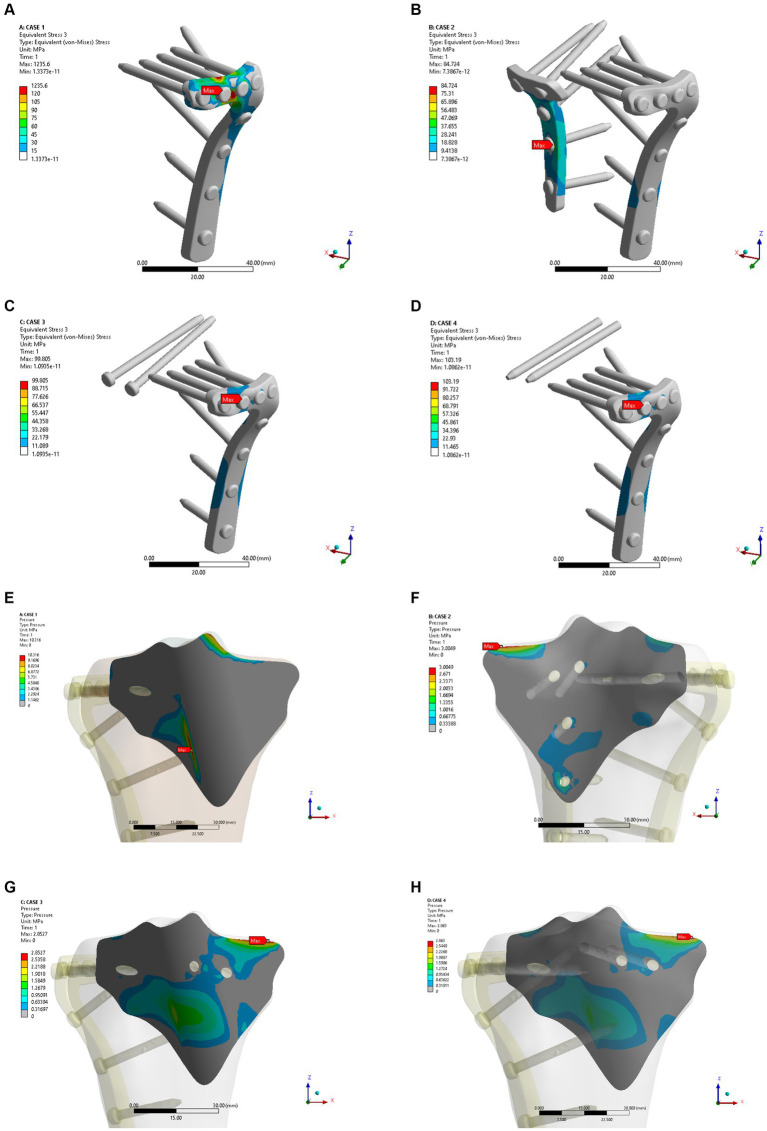
Maximum stress **(A–D)** and contact stress **(E–H)** in the four internal fixation models of the normal bone group.

As shown in Figures 4A–D, the fixation method of Model A showed the most unstable scenario, with maximum displacement occurring when the fracture was fixed with a lateral L-plate alone in the normal bone group. The maximal displacement under the described load was 0.60032 mm, and the area of maximum displacement appeared along the margin of the posteromedial fracture line. With the addition of posterior tibial plate fixation in Model B, the maximum displacement was reduced to 0.38882 mm, which is approximately one-third less than in Model A. The maximum displacement area of Model B appeared near the intercondylar eminence of the tibial plateau. In Model C (addition of two cannulated screws to fix the posterior fracture block), the stability was improved compared to Model A and the maximum displacement was reduced to 0.42345 mm. In Model D, the maximum displacement of two cannulated screws fixed from the front of the fracture block was found to reach 0.42273 mm, which showed no significant difference compared with Model C. In the normal bone model, Figures 4E–H showed the sliding distances of the fracture surfaces in the four models, in which the sliding distance of Model A was 0.05442 mm, and the sliding distances of fracture surfaces of models B, C and D were 0.00194 mm, 0.01341 mm and 0.01361 mm, respectively. Figures 5A–D presents the stress (von Mises) of the implants under four different fixation methods. The maximum stress of Model A was 1235.6 MPa, with the screws located below the articular surface playing an essential role in the fixation of the posterolateral fracture block. The maximum stress in Model B was 84.724 MPa, which occurred at the position of the posterior tibial plate. The maximum stress of Model C and Model D were 99.805 MPa and 103.19 MPa, respectively, and the maximum stress occurred at similar sites. As shown in Figures 5E–H, the corresponding contact stresses on the fracture surfaces of the four models in the normal bone model were 10.316 MPa, 3.0049 MPa, 2.8527 MPa and 2.863 MPa, respectively.

**Figure 6:**
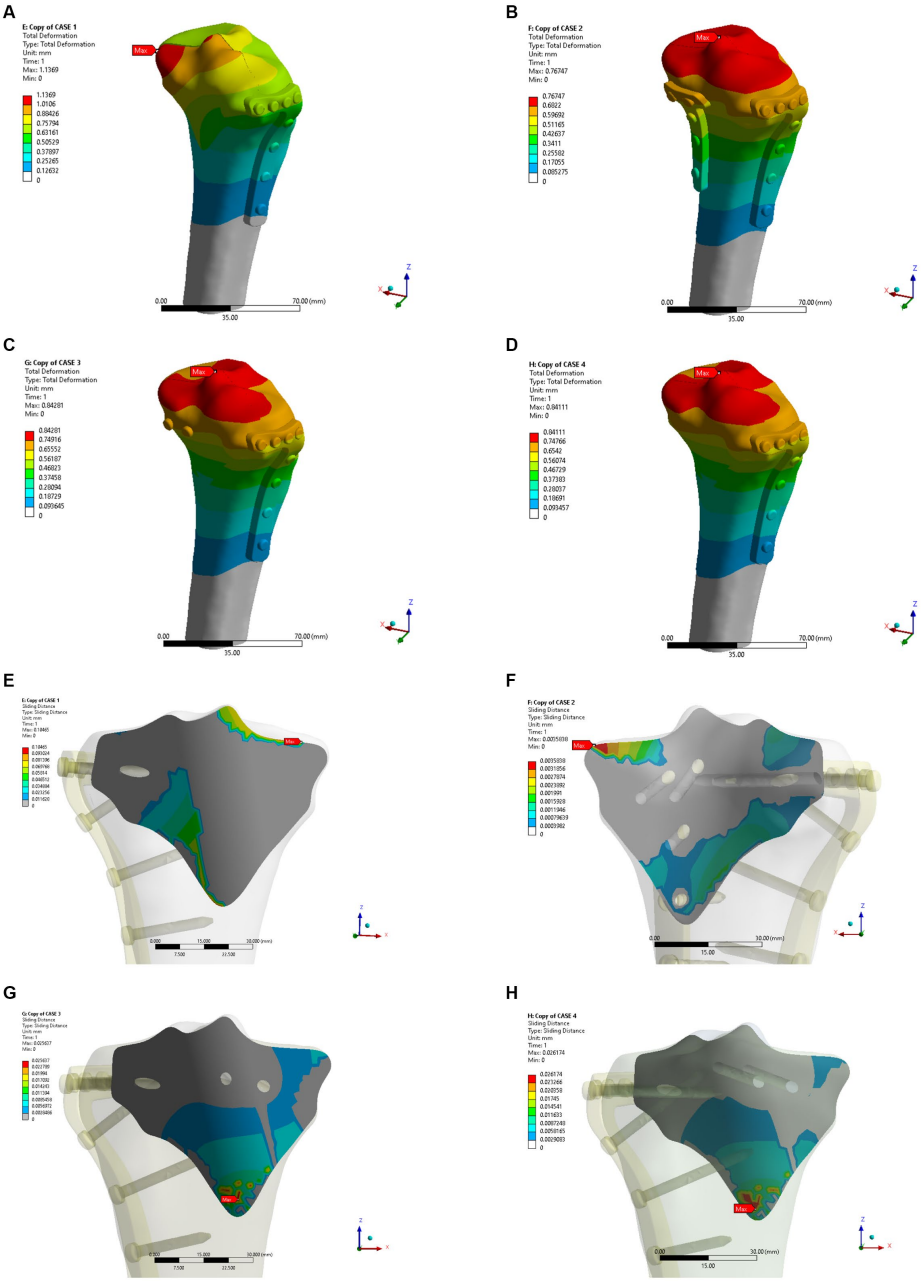
Maximum displacement **(A–D)** and interface displacement **(E–H)** in the four internal fixation models of the osteoporosis group.

**Figure 7:**
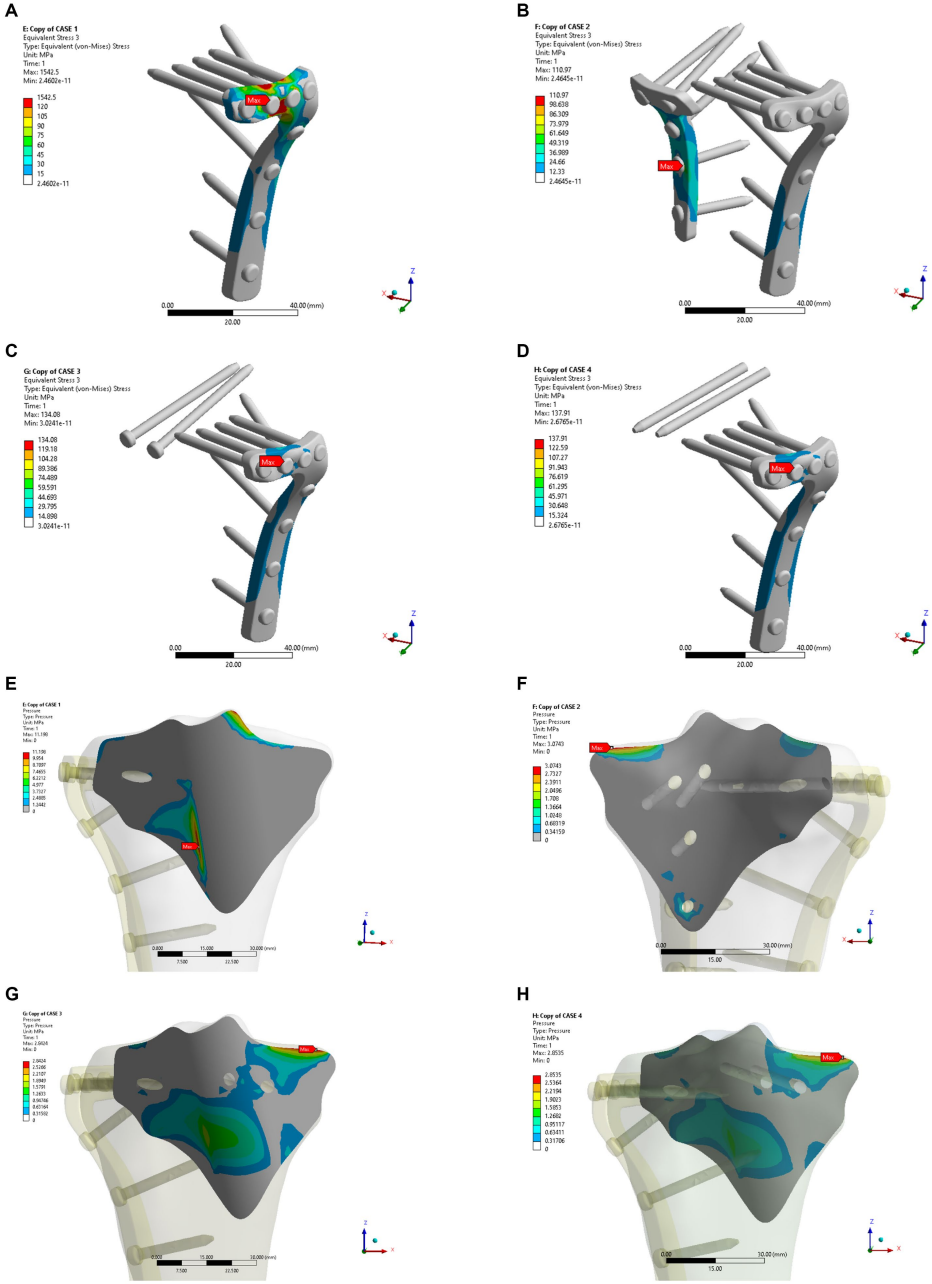
Maximum stress **(A–D)** and contact stress **(E–H)** in the four internal fixation models of the osteoporosis group.

**Figure 8:**
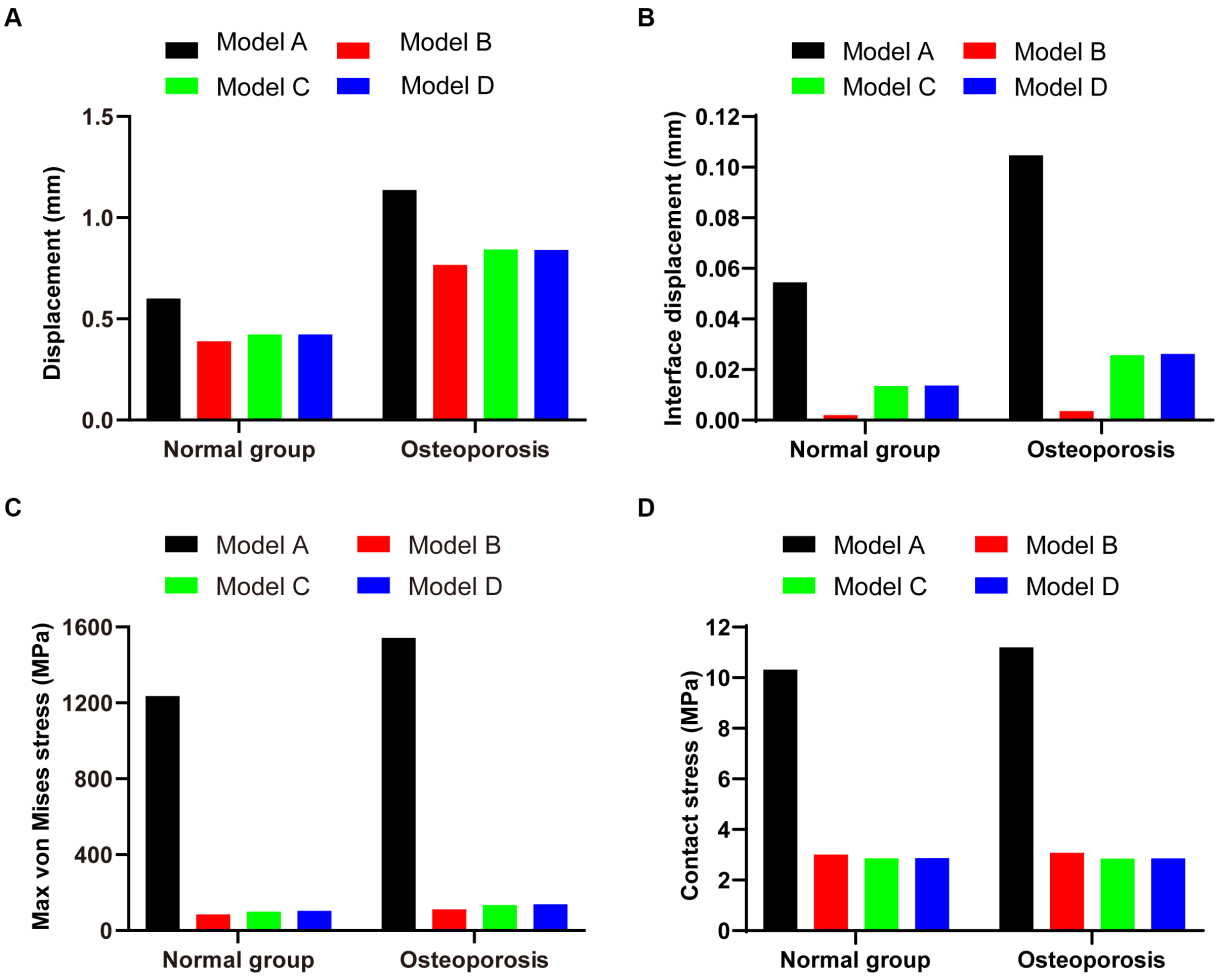
**(A,B)** Maximum displacement and interface displacement in the four internal fixation models of the normal and osteoporosis groups. **(C,D)** Maximum stress and contact stress in the four internal fixation models of the normal and osteoporosis groups.

In the osteoporosis models, the maximum displacement was 1.1369 mm for Model A, 0.76747 mm for Model B, 0.84281 mm for Model C, and 0.84111 mm for Model D (Figures 6A–D). As shown in Figures 6E–H, the corresponding sliding distances on the fracture surfaces of the four models were 0.10465 mm, 0.00358 mm, 0.02563 mm, and 0.02617 mm, respectively. In the osteoporosis models, the maximum stress of models A, B, C, and D reached 1542.5 MPa, 110.97 MPa, 134.08 MPa, and 137.91 MPa, respectively (Figures 7A–D). Accordingly, the maximum stress increased by about one-third compared to the stress measured in the normal bone model. Figures 7E–H showed the contact stresses of the fracture surfaces in the four models, of which the contact stress of Model A was 11.198 MPa, and the contact stresses of the fracture surfaces of models B, C, and D were 3.0743 MPa, 2.8424 MPa, and 2.8535 MPa, respectively. Figure 8A illustrates the comparison of the maximum displacement between the simulated normal bone model and the osteoporotic bone model. Notably, the analysis of the maximum displacement in the osteoporotic bone model revealed a trend similar to that of normal bone. Yet, in the osteoporosis model, the maximum displacement of each group was about twice that of the equivalent normal bone model. It is also shown that the trend of interfacial displacement in the normal and osteoporotic groups was consistent (Figure 8B). As shown in Figure 8C, the simulation results of the implants in the normal and osteoporotic bone models showed that the maximum stress of Model A was significantly higher than that of the other three groups. In addition, the trends of contact stress changes were similar for the normal bone model and the osteoporosis model (Figure 8D).

In conclusion, compared with the other three fixation models, Model B exhibited the lowest displacement and stress and can, therefore, be assumed to be the optimal internal fixation for Hoffa-like fracture of the tibial plateau model.

## 4. Discussion

Most clinical cases of tibial plateau Hoffa-like fractures are caused by high-energy trauma, such as a fall from a height or a motor vehicle accident. The fracture is typically managed with a lateral or posterior surgical approach for open reduction and internal fixation (ORIF) (1). In 2009, Luo et al. proposed a CT-based tibial plateau fracture staging method known as “three-column staging,” offering two main advantages (8): First, the 3D reconstruction of the CT images allows for the inclusion of posterior fractures not captured by the Schatzker staging and the AO/ATO staging, thereby facilitating a more comprehensive evaluation of the fracture damage in the tibial plateau. Second, the three-column staging helps the surgeon to develop a reasonable surgical approach, especially for the accurate and effective fixation of the posterior fracture block. Notably, based on the concept of three-column staging, Chang et al. proposed an extended four-column staging theory by further sub-dividing posterior tibial plateau fractures into posterior medial and posterior lateral fractures (17, 18). The fracture model selected in this study is highly representative, as it takes into account both split fractures of the medial tibial plateau and collapsed fractures of the lateral side. To date, a superior fixation method for Hoffa-like fractures of the tibial plateau remains to be identified. The herein presented results shed light on substantial differences in the stability of various fixation methods. Under the present conditions, the fixation stability of Hoffa-like fractures of the tibial plateau can be improved by adding posterior medial plate fixation to fixation with the lateral splint. Moreover, supplementary fixation of the fracture with hollow screws also showed increased fixation stability as compared to the isolated use of the lateral splint.

Theoretically, for the majority of fractures with minor displacement, the posteromedial approach with plate internal fixation can be considered the most appropriate treatment option (3). When the fracture is too posterior in the coronal plane and the displaced fracture fragment is too small, a posterior medial approach may be adequate. Yet, this procedure is known to increase trauma (19). In the FEM study, exceeding the capacity of the material properties would be deemed as failure of fracture fixation (20–22). In clinical reality, it is widely assumed that—if internal fixation of a tibial plateau fracture is deemed suboptimal—the patient must be instructed to perform severely limited weight bearing and/or limited range of knee motion. Otherwise, patients’ non-compliance may echo in the deformation of the internal fixations or re-displacement of the fracture. Excessive displacement of the implants can lead to loosening of the screws and compromise the stability of the internal fixation, thereby increasing the risk of fixation failure (23, 24). The results of the FEM in this study revealed that displacement was highest in Model A and lowest in Model B—with both the healthy bone model and the osteoporotic model. Therefore, in cases of posterior column fractures with accompanying tibial plateau involvement, additional posterior lateral plate fixation is required to withstand the load exerted by body weight, a consideration particularly important in patients with osteoporosis.

When simulating the physiological loading conditions, we referred to previous studies (16, 25). In fact, prior research has shown that the posterior load on the tibial plateau is highest during knee flexion, whereas the stress on the posterior column of the tibial plateau remains relatively low when the knee is extended (26–28). In this study, an axial load of 2,500 N was chosen, with 2,500 N being considered the limiting load—partitioned into 60% medial and 40% lateral. Of note, this load and ratio are in line with FEM studies reported in the available literature (25, 29). Regarding the bone characteristics, we followed the Young’s modulus of cortical and cancellous bone described in previous FEM studies (14, 30–32). In most studies, cortical bone was set to about 17 GPa, while the parameters regarding cancellous bone were more variable, which is consistent with clinical experience. In this study, we chose 430 MPa as the Young’s modulus of cancellous bone.

In terms of resistance to axial loading, Zeng et al. demonstrated that the fixation of the posterior internal T-shaped support plate is stronger than that of the parallel tension screws, which agrees with the findings of our analysis (33). Further fixation methods for the treatment of tibial plateau fractures with coronal splits have been reported with promising outcomes. Notably, these studies consistently conclude that adequate fixation of the posterior medial splinter is essential (4, 34). Vice versa, Samsami et al. analyzed the effect of a posterior medial split bone mass in their model and found that a single posterolateral plate fixation was not appropriate (35). In this context it is worth mentioning that a wide array of confounding variables such as the fracture type, the plate design, or the screw diameter may affect the final fixation outcome. Future studies are needed to address this variance.

Nevertheless, as a possible influencing factor, we investigated the stability of medial split fractures after fixation with hollow screws from different positions in Model C and Model D. The analysis revealed a clear improvement in stability compared with Model A (without hollow screw fixation). Interestingly, no significant difference between Model C and Model D was observed. This finding suggests that the difference in medial fractures after hollow screw fixation from the front or back of the tibia is not significant. Hence, the use of additional hollow screw fixation can be considered a simple and reliable method to ensure stable fracture fixation whilst avoiding unnecessary trauma due to intraoperative over dissection.

Bone mass is also a relevant determinant possibly affecting biomechanical analyses. In fact, patients with clinically diagnosed osteoporosis are more likely to experience postoperative failure of internal fixation (36, 37). The elastic modulus of cortical and cancellous bone in the model were modified not only according to normal but also osteoporotic bone architecture. The results of this sub-analysis showed, that in the case of osteoporosis, greater displacement and higher stresses occur in the fracture block. Therefore, based on the findings of the FEM analysis in this study, the concept of lateral and posterior plate fixation in Model B seems to be more effective in osteoporotic bone. When posterior lateral plate fixation is applied, the effect of low bone mass appears to be less important, although it may increase the damage caused by the procedure itself. In clinical practice, posterior lateral plates for the tibial plateau are commonly used as reconstructive plates or T-plates. The results of our analysis indicate that additional internal fixation with a posterior anti-gliding plate behind the tibial plateau is superior for Hoffa-like fractures in osteoporotic patients. Studies investigating unstable posterior medial tibial plateau fracture blocks have shown that slight knee flexion can cause significant fracture instability, thus highlighting the specificity of the treatment for this rare and easily overlooked fracture type (38, 39). By conducting this FEM analysis, we aimed to contribute to the treatment optimization of posterior column fractures involving the tibial plateau from a biomechanical and clinical-translational perspective. Herein, we report on the value of the additional fixation of posterior lateral plate in Hoffa-like fractures affecting the tibial plateau.

However, this study needs to be interpreted in light of its inherent limitations. In clinical practice, screw cut-out is one of the crucial contributors of fixation failure, a variable not considered in this study. As mentioned above, only two different bone masses were included in our analyses. In addition, the fracture type, loading situation, and screw size were set to a single setting. The purpose of this study was to investigate the effect of different surgical fixation modalities on the fixation outcomes of the described fracture types. However, the simulated fracture model did not take into account the soft tissues surrounding the knee joint including neurovascular, meniscal, and ligamentous tissues. Accordingly, the effect of these tissues on the results of the FEM analysis needs to be deciphered in future studies. As a result, the reported absolute values may be questionable. Yet, the relative differences between the various scenarios and the association of the main effects remain relevant. The results of this study will provide a reference for future biomechanical evaluation of Hoffa-like tibial plateau fractures and the loading of varying axial loads as well as for the investigation of other rare fracture types or unique fracture fixation methods.

## 5. Conclusion

Our results suggest that lateral plate combined with posterior plate fixation is superior for Hoffa-like tibial plateau fractures involving the posterior column and may be also superior for the treatment of posterior-lateral collapsed fractures of the proximal tibia.

## Data availability statement

The original contributions presented in the study are included in the article/supplementary material, further inquiries can be directed to the corresponding author.

## Ethics statement

The studies involving human participants were reviewed and approved by the Ethics Committee of the Wuhan Union Hospital. The patients/participants provided their written informed consent to participate in this study.

## Author contributions

HX and JD designed the study and wrote the initial draft of this manuscript. ZZ performed the data collection and statistical analyses. SK and AP searched and selected relevant studies. LK and BM put forward valuable opinions on the subject design. ML, GD, and GL critically reviewed and approved the final manuscript. All authors contributed to the article and approved the submitted version.

## Funding

This work was supported by the National Natural Science Foundation of China (No. 82202676), China Postdoctoral Science Foundation (No. 2021 M701333), and the Hubei Province Key Laboratory of Oral and Maxillofacial Development and Regeneration (No. 2021kqhm007).

## Conflict of interest

The authors declare that the research was conducted in the absence of any commercial or financial relationships that could be construed as a potential conflict of interest.

## Publisher’s note

All claims expressed in this article are solely those of the authors and do not necessarily represent those of their affiliated organizations, or those of the publisher, the editors and the reviewers. Any product that may be evaluated in this article, or claim that may be made by its manufacturer, is not guaranteed or endorsed by the publisher.
